# Rodent Models of Non-classical Progesterone Action Regulating Ovulation

**DOI:** 10.3389/fendo.2017.00165

**Published:** 2017-07-24

**Authors:** Melinda A. Mittelman-Smith, Lauren M. Rudolph, Margaret A. Mohr, Paul E. Micevych

**Affiliations:** ^1^Department of Neurobiology, David Geffen School of Medicine at UCLA, The Laboratory of Neuroendocrinology, Brain Research Institute, University of California Los Angeles, Los Angeles, CA, United States

**Keywords:** estrogen, progesterone, reproduction, lordosis, ovulation, luteinizing hormone, hypothalamus, kisspeptin

## Abstract

It is becoming clear that steroid hormones act not only by binding to nuclear receptors that associate with specific response elements in the nucleus but also by binding to receptors on the cell membrane. In this newly discovered manner, steroid hormones can initiate intracellular signaling cascades which elicit rapid effects such as release of internal calcium stores and activation of kinases. We have learned much about the translocation and signaling of steroid hormone receptors from investigations into estrogen receptor α, which can be trafficked to, and signal from, the cell membrane. It is now clear that progesterone (P4) can also elicit effects that cannot be exclusively explained by transcriptional changes. Similar to E2 and its receptors, P4 can initiate signaling at the cell membrane, both through progesterone receptor and via a host of newly discovered membrane receptors (e.g., membrane progesterone receptors, progesterone receptor membrane components). This review discusses the parallels between neurotransmitter-like E2 action and the more recently investigated non-classical P4 signaling, in the context of reproductive behaviors in the rodent.

## Introduction

For years, we understood that steroids functioned as ligand-gated transcription factors that enacted changes in gene expression (1). This classic dogma of steroid hormones operating exclusively through nuclear response elements to modify gene transcription has been challenged and expanded. While many processes do rely on this “classical” mode of steroid hormone signaling, our understanding of how steroid hormones signal has developed dramatically, including a recent argument for steroid classification as neurotransmitters (2, 3). Much of this recent understanding of how steroids signal was developed from studies about estrogen (E2) signaling, but we now understand that non-classical signaling is likely to occur across all classes of steroids. This review briefly highlights initial discoveries of the molecular methods of non-classical E2 signaling and focuses on more recent advances in our understanding of how progesterone (P4) signaling appears to function in a similar fashion, with membrane-initiated events involving a variety of receptors that regulate critical aspects of reproduction.

First, the nomenclature used to discuss steroid hormone action should be considered. The “non-classical” method of steroid action has been called *rapid* or sometimes *non-nuclear*. Importantly, caution should be exercised when generalizing these terminologies to encompass all “non-classical” steroid signaling. First, the term *rapid* is relative and does not indicate a clear temporal delineation by which effects could be categorized as *non-rapid*. At what time point, do steroid effects cease to be *rapid*? In this review, we will use the term *rapid* to describe effects occurring within minutes, though throughout scientific literature this term may be used more loosely. Second, while steroid effects can be mediated by non-nuclear receptors, in certain cases, it may be technically incorrect to refer to these events as *non-nuclear*. Activation of membrane-localized receptors can (and often do) ultimately lead to activation of nuclear response elements and transcriptional changes [e.g., *via* activation of CREB (4, 5)]. Therefore, it is more accurate to use terms such as *non-classical* or *membrane-initiated signaling* to encompass all signaling arising from steroid hormone activation of membrane-bound receptors. This inclusive terminology is appropriate for non-classical steroid signaling of all varieties, as it describes not only initiation of rapid second messenger pathways but also effects arising from the membrane and culminating in transcriptional and translational changes.

Over the past several decades, E2-activated non-classical signaling cascades have been elucidated by research groups, including our own, who were largely focused on the mechanisms of E2 signaling [reviewed in Ref. (6–11)]. Relatively little is known about membrane-initiated effects of P4 (particularly in the CNS), but there are striking similarities between non-classical E2 and P4 signaling, suggesting a commonality of mechanisms. This review will discuss the hallmarks of non-classical E2 signaling alongside the growing literature of membrane-initiated P4 signaling, and how neurotransmitter-like effects of these steroid hormones function, often interdependently, to direct the structure and function of neuroendocrine systems critical for reproduction.

## Non-Classical Steroid Signaling

While the critical role of steroid hormones in reproduction is well established, new lines of research are demonstrating that the formerly termed “gonadal hormones” are also synthesized and act at extra-gonadal regions [reviewed in Ref. (3, 12, 13)]. Non-classical steroid signaling has been most thoroughly studied with E2; however, as novel mechanisms of steroid signaling continue to be the target of investigation, it appears that most (and perhaps all) steroid hormones can and do function as E2 does, in both classical and non-classical ways. To demonstrate the parallels between the known aspect of non-classical E2 signaling and the developing literature of neurotransmitter-like P4 signaling, this review is structured to address the aspects of P4 signaling that are known to replicate the mechanisms of neurotransmitter-like E2 signaling. Therefore, we focus on rapid, membrane-initiated effects of P4, neural synthesis of P4, and the wide array of P4-binding proteins and their trafficking, all in the greater context of the classical reproductive events of the female rodent: lordosis and the luteinizing hormone (LH) surge triggering ovulation.

### Rapid, Membrane-Initiated Effects

#### Estradiol

Hypothalamic circuits are essential regulators of negative and positive feedback loops that govern reproductive functions. These circuits are exquisitely sensitive to and regulated by steroid hormones. While this review focuses on the mechanisms of P4-mediated signaling in reproduction, it is important to consider the interaction of P4 and E2 signaling pathways and how these steroid hormones act to co-regulate reproductive events in animals, and specifically in female rodents.

Initial evidence indicating non-genomic actions of E2 has been known for decades, as effects could be elicited on a time course too short to allow for transcription and translation to occur (14). Most research during this time assessed steroid hormone effects hours or days after hormone treatment, precluding the possibility of discovering rapid effects and further solidifying the idea that steroid hormones acted to regulate transcription. Since then, examination of additional experimental time points and new methodologies have allowed for the assessment of rapid effects of E2 and P4 on neural structure, function, and behaviors. With a short exposure to E2, mu-opioid receptors are activated and internalized, a step critical for the subsequent, delayed expression of sexual receptivity (15). This effect can be elicited within minutes by cell-impermeable E-6-BSA (E2 conjugated to bovine serum albumin), demonstrating that these E2 actions are both rapid and membrane-initiated (16). *In vitro*, membrane-initiated E2 signaling elicits changes in gene expression (17), demonstrating that E2 action at the membrane does not preclude changes in gene expression, and can initiate genomic changes. Indeed, our own *in vitro* experiments have demonstrated mRNA and protein changes occurring 24–48 h after E-6-BSA treatment (18). *In vivo*, membrane-initiated E2 signaling can augment the effects of “classical” E2 signaling, the latter involving genomic mechanisms (19). This non-classical E2 signaling at the membrane occurs *via* estrogen receptor α (ERα) coupled to the metabotropic glutamate receptor 1a (mGluR1a) at the cell membrane, initiating second messenger intracellular signaling cascades (5, 20). Further highlighting the interaction of non-classical and classical steroid signaling, ERα signaling at the membrane is secondary to the trafficking of “classical” ERα to the cell surface. Here, ERα associates with caveolin molecules that are necessary for membrane-initiated E2 signaling *via* mGluR1a (21). Palmitoylation sequences in the ERα gene provide a substrate for association with caveolin-1 (22), which mediates trafficking to the cell membrane and mGluR-associated signaling (23). Therefore, both membrane-initiated and classical genomic E2 signaling is mediated by ERα, and it is its cellular localization that determines which mechanism of signaling is activated. It is possible, but not yet elucidated whether P4 acts like E2, using the same receptor to mediate both classical and membrane-initiated effects *via* trafficking of the receptor.

#### Progesterone

Accumulating evidence suggests that P4, similar to E2, can signal through non-classical mechanism(s): P4 or membrane-restricted P4 (P4-3-BSA) affects cellular signaling in seconds to minutes, suggesting a plasma membrane-initiated action. In the CNS, rapid actions of P4 have been shown to alter neuronal responsivity in various cell types and affect a variety of physiological processes, including neuroprotection and reproduction (24–30). For example, in ovariectomized (ovx) rats, within 5–15 min after P4 administration, responses to glutamate decrease, and inhibitory responses to GABA increase (25). Relevant to reproduction, P4 upregulates oxytocin receptor binding and lordosis behavior within 30 min of administration (24). Rapid effects of P4 also modulate reproduction-related neurotransmitter release that can alter sexual receptivity, such as dopamine and acetylcholine (31), as well as norepinephrine-stimulated cyclic AMP (cAMP) (32, 33). P4 membrane signaling has been associated with gonadotropin-releasing hormone (GnRH) release, although the exact role of P4 in GnRH release is unresolved (29, 34). Using a superfusion technique, P4-3-BSA stimulates GnRH release, demonstrating that P4-induced GnRH release is initiated at the plasma membrane (34). Given that P4 regulates neuroendocrine events that occur on both acute and more protracted time scales, it follows that the effects of P4 could be mediated through both classical and non-classical signaling mechanisms. A growing body of literature indicates that P4 metabolites can also function in various physiological pathways having anti-anxiety, anesthetic, and neuroprotective properties [e.g., Ref. (35–37)]. However, whether these metabolites signal through classical or non-classical receptors or whether their signaling is membrane-initiated or nuclear is not yet known.

### Non-Classical Receptor Signaling

Originally, there was only one known estrogen receptor, “ER” (now called ERα). Since the subsequent discovery of ERβ, there is an ever increasing number of estrogen-binding proteins that have been discovered. In addition to ERα and ERβ, there has been a growing literature on mechanisms of E2 mediated *via* non-classical receptors, such as the G protein-coupled estrogen receptor 1, GPER (originally GPR30). Other estrogen receptors, putative and known, including ER-X and STX, remain the targets of many lines of research in the non-classical E2 signaling field. The continual discovery of novel receptors suggests that P4 signaling will also involve a variety of P4-binding proteins through which non-classical P4 action occurs.

### “Classical” Progesterone Receptor (PGR) on the Membrane

**Figure 1:**
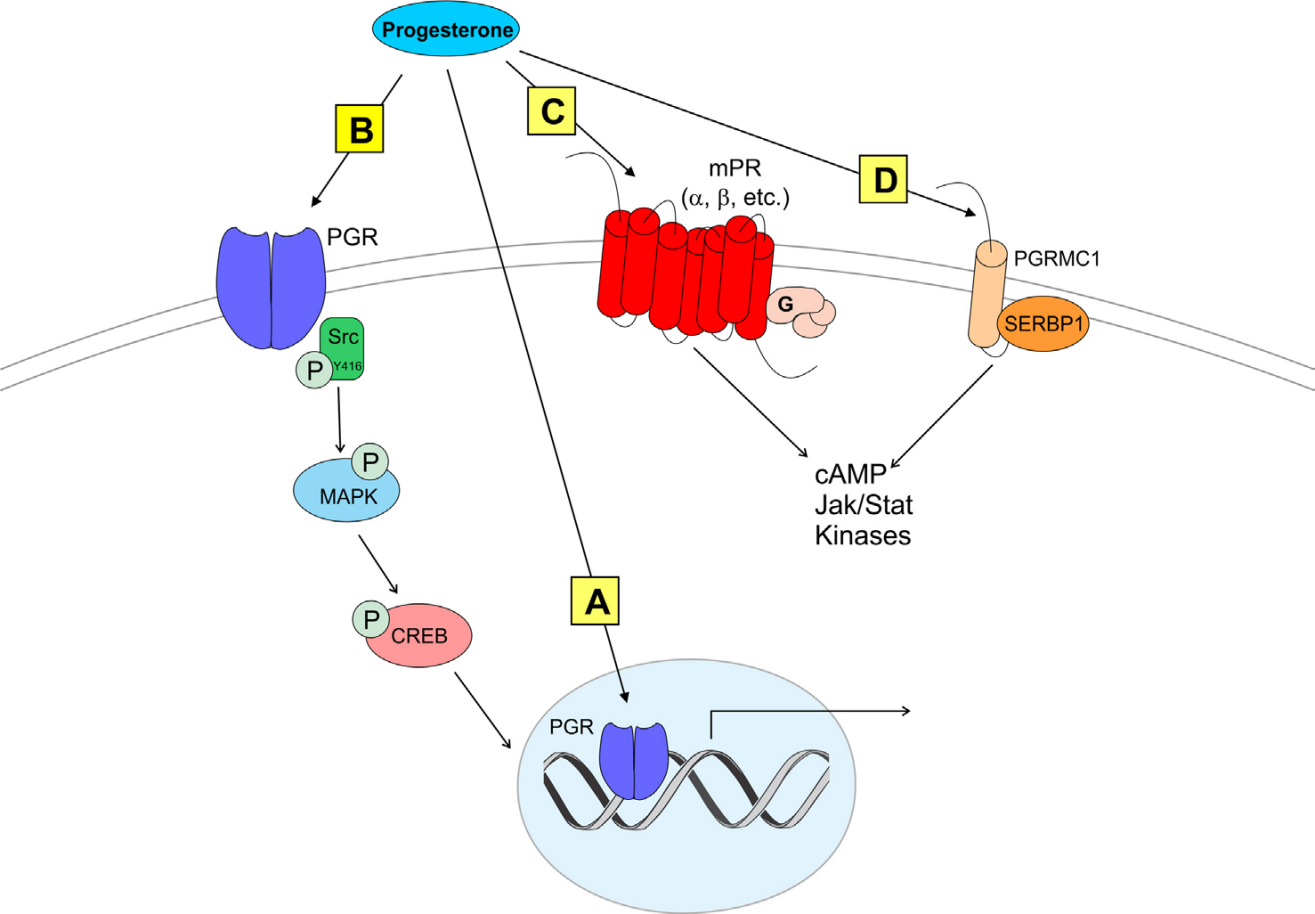
Modes of progesterone signaling in the rodent. Classical progesterone receptor (PGR) can mediate progesterone signaling classically **(A)**, by binding to DNA progesterone response elements. PGR can also be trafficked to the plasma membrane [as in panel **(B)**] where it can activate rapid intracellular signaling cascades involving kinases such as Src (41–43). It is unknown whether membrane PGR transactivates another receptor like an mGluR as estrogen receptors have been shown to do (5, 16, 44). Multiple novel membrane progesterone receptors (mPRs) have been recently discovered and described, such as mPRs α, β, δ, and γ **(C)**. mPRs can activate signaling cascades *via* G proteins, which go on to affect cyclic AMP (cAMP) pathways (45, 46). Finally, progestins can bind to progesterone receptor membrane component 1 [PGRMC1 **(D)**]. PGRMC1 can work in concert with SERBP1 to affect cAMP, Jak/Stat, and multiple kinase pathways [reviewed in Ref. (47)].

Similar to E2 signaling, we now understand that not only does P4 signaling involve novel receptors but also that classical receptors can function in novel ways (e.g., at the membrane). The “classical” PGR has many structural properties similar to ERα that could allow membrane trafficking (38). For example, PGR contains a 9 amino acid motif that mediates palmitoylation-induced membrane translocation (38). This palmitoylation sequence facilitates association of mGluRs with ERs and appears to be conserved across multiple steroid hormone receptors, including PGR. Therefore, P4 could act as E2 does at the membrane, *via* palmitoylation-induced trafficking and association with mGluRs. However, whether there is a PGR–mGluR association has not been examined to date. Though not discussed in this review, there is also evidence that PGR can be activated in a “ligand-independent” manner by neurotransmitters and other factors to affect various processes and behaviors critical to reproduction [reviewed in Ref. (39, 40)]. In addition to PGR, other, non-classical P4-binding proteins have been more recently discovered that may contribute to reproductive processes (see Figure 1).

#### Membrane Progesterone Receptors (mPRs)

The wealth of data suggests that classical PGR is critical for P4 facilitation of the LH surge, but LH release *can* be affected in PGR knockout (PRKO) animals, suggesting the possibility that P4 signals through non-classical PGRs to affect LH release (29). While the specific roles of non-classical PGRs in LH release are unknown, their neuroanatomical locations and their functions in non-neural reproductive tissues supports the idea that these receptors can mediate rapid, membrane-initiated P4 signaling. Two different types of non-classical receptor families mediate membrane-initiated actions of P4: the 7 transmembrane domain membrane P4 receptors (mPRs) that belong to the Class II progestin and adipoQ receptor (PAQR) family (45, 46) and the membrane-associated PGR family including progesterone receptor membrane components 1 and 2 [PGRMC1 and 2 (46, 48); discussed below]. There are various subtypes of mPR: mPRα (PAQR7), mPRβ (PAQR8), mPRγ (PAQR5), mPRδ (PAQR6), and mPRε (PAQR9) (49). These mPR subtypes exhibit high-affinity binding of P4 at the cell membrane (49, 50) and act somewhat like unique G protein-coupled receptors (51). Some mPRs (including mPRα, mPRβ, and mPRγ) can mediate rapid P4 actions by activation of an inhibitory G protein (G_i_) and suppression of adenylyl cyclase activity and cAMP production [Figure 1; (45, 46)].

Based on expression patterns and responsivity to E2, the most reproductively relevant mPRs appear to be mPRα and mPRβ. mPRα expression levels are high in the testis, ovary, and placenta (45). Expression of mPRα is also observed, albeit at lower levels, in the CNS, including the hypothalamus (52, 53). Low central expression may indicate that mPRα mediates rapid P4 effects more so in the periphery than in the CNS. Centrally, mPRβ is the primary mPR subtype (45), with particularly high expression in reproductively relevant hypothalamic structures including the paraventricular nucleus, ventromedial hypothalamus, and arcuate nucleus, as well as forebrain structures including the medial septum and horizontal diagonal band (53). One of the first demonstrations of P4 signaling through mPRs showed that progestin-dependent mPRα activation increases sperm motility and oocyte maturation in teleost fish (45, 54, 55). While both mPRα and mPRβ mRNAs are demonstrated in the hypothalamus, their physiological relevance has not been determined in the rodent. In addition to localization patterns, E2 responsivity of mPR subtypes is also isoform specific. E2 does not appear to affect hypothalamic levels of mPRα (53), while on the other hand, mPRβ is induced by E2 in the female rat hypothalamus *in vivo*. However, *in vitro*, E2 did not induce upregulation of mPRβ in immortalized RP3V kisspeptin (Kiss1) neurons (41), suggesting that this upregulation may occur in cell types not directly involved in governing the LH surge (discussed below). However, local expression of mPRs, along with mPRβ induction by E2, indicates mPRs could function in a facilitatory role in signaling pathways underlying the surge.

#### Progesterone Receptor Membrane Components (PGRMCs)

Progesterone receptor membrane components are another set of non-classical proteins that mediate P4 membrane-initiated signaling. PGRMCs are thought to be involved in an array of functions, including trafficking of other receptors to the membrane, cell survival, modulation of enzymes involved in steroid synthesis, steroidogenesis, and progesterone-responsiveness (48, 56–58). Most work investigating PGRMC function stems from cancer biology research and involves non-neural reproductive tissues, so the question remains whether PGRMCs play similar roles in the brain. In fact, only PGRMC1 (also known as 25-DX in the rodent) appears to bind progestins (56, 59) and a functional role for PGRMC2 has yet to be elucidated. However, PGRMC1 and PGRMC2 are expressed in both peripheral reproductive tissues and throughout reproductively relevant brain nuclei, particularly in the hypothalamus. Therefore, while the functionality of these receptors in reproduction is not fully understood, their expression patterns suggest they are anatomically positioned for a role in reproduction (52, 60).

Progesterone receptor membrane component 1 appears to function primarily through partnering with other proteins. In the ovary, PGRMC1 forms a complex with serpine mRNA-binding protein 1 (SERBP1), which is necessary for P4’s antiapoptotic effect in granulosa cells (56, 61). In the brain, patterns of PGRMC1 and SERBP1 expression strongly overlap, further supporting the idea that these proteins partner to form a functional receptor (52, 61). Interestingly, the region of the brain containing the highest expression of mRNAs encoding PGRMCs and SERBP1 is the anteroventral periventricular nucleus (AVPV) (52), one of the most important neural sites for estrogen positive feedback underlying ovulation. Indeed, ovarian hormones, alone or in concert, regulate PGRMC1, SERBP1, PGRMC2, and mPRβ expression in the brain (53, 62). PGRMC1 and SERBP1, but not mPRs, are expressed in GnRH neurons and have been implicated in P4’s inhibition of GnRH neuronal activity through protein kinase G signaling (30), suggesting a possible role for PGRMC1 in terminating the LH surge. PGRMC1 may also function as an adaptor protein for multiple classes of steroid receptors, as PGRMC1 has been shown to transport mPRα and ERβ to the cell surface in a breast cancer cell line (63), but whether PGRMC1 acts as an adaptor protein in the brain is unknown.

The PGRMC structure is different than that of G protein-coupled receptors or of classical steroid hormone receptors. PGRMCs are unique in that they possess a cytochrome b5-like heme/steroid-binding domain where enzymes involved in steroid hormone synthesis can bind (64). This binding domain allows PGRMC1 to bind and enhance the activity of steroid-synthesizing P450 enzymes (38, 48). For example, it has been demonstrated that PGRMC1 binds to Cyp51A1 and is required for its activity in cholesterol synthesis (38). PGRMC1 also promotes the activity of aromatase, an enzyme crucial for estrogen synthesis (65). These interactions with P450 enzymes suggest a role for PGRMC1 in steroid synthesis, and perhaps PGRMC1 is involved in neural synthesis of steroid hormones.

### Neural Synthesis of Steroids

A hallmark of “non-classical” steroid action is the understanding that steroids can be synthesized on demand, and in neural tissue. This contradicts the long-standing dogma of peripheral steroid synthesis and transport to distant target tissues was the sole mechanism of steroid hormone action. Indeed, the notion of neurosteroid production has been known for years, in rodents and in avian species [for review, see Ref. (66, 67)], but we now better understand the mechanisms of how this occurs. In birds and rodents, local action of aromatase in the brain is responsible for the conversion of testosterone to E2, allowing for on-demand production of E2 (68), which has rapid, potent effects on seizure behavior (69), learning and memory (70), and reproduction-related functions (71), in both males and females (72). Given that highly specific enzymatic activity appears to be responsible for rapid, local production of E2 in the nervous system, it is likely that P4 synthesis in the brain is regulated in a similar fashion. Indeed, it is known that P4 is produced from steroid precursors within specific regions and cell types of the nervous system, but the mechanisms of this process, including spatiotemporal resolution and rate-limiting step of this pathway have yet to be determined. However, given the parallels between non-classical E2 and P4 signaling, it is likely that the regulation of P4 production in the nervous system is governed by similar mechanisms.

#### Neuroprogesterone

**Figure 2:**
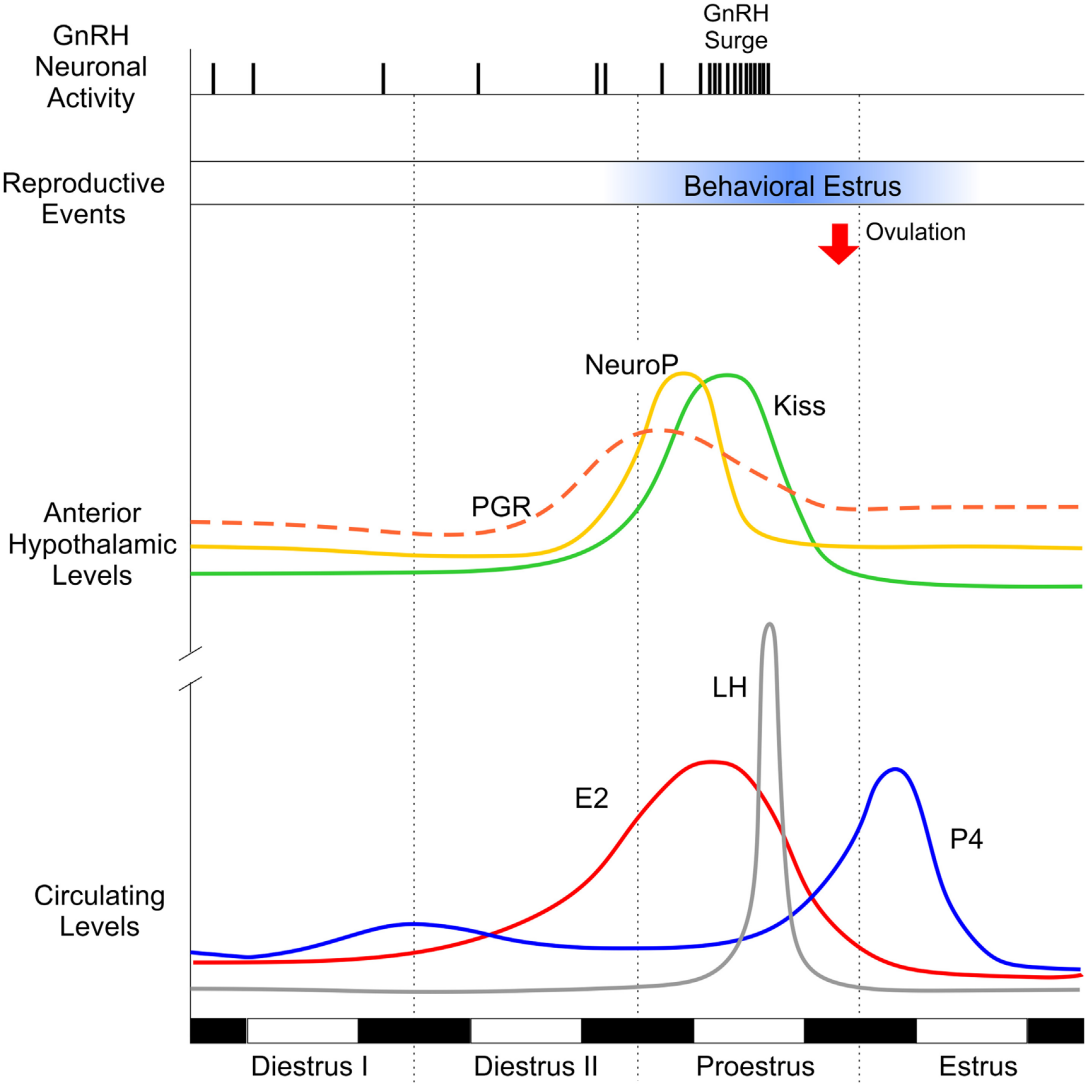
Key factors underlying ovulation and sexual behavior in the female rodent across the rodent estrous cycle. Dark bars represent night time; light bars represent daytime. Diestrus I and II are marked by low levels of circulating (lower panel) hormones, such as estradiol (E2, red line), progesterone (P4, blue line), and luteinizing hormone (LH, gray line). Similarly, hypothalamic levels of P4 (neuroP, yellow line), progesterone receptor (PGR, orange dashed line), and kisspeptin (Kiss1, green line) are relatively low. Circulating E2 levels begin to rise (secondary to the development of ovarian follicles), triggering upregulation of PGR and Kiss1 in the anterior hypothalamus. E2 peaks on proestrus, inducing rapid synthesis of neuroP in hypothalamic astrocytes. In addition to the stimulatory effects of E2, the rise in local hypothalamic levels of neuroP augments Kiss1 expression, culminating in a gonadotropin-releasing hormone (GnRH) surge, which triggers surge release of LH from the pituitary and ovulation (red arrow). Note that peripheral P4 does not peak until after the LH surge triggering ovulation, supporting the idea that the critical source of preovulatory P4 is hypothalamic (neuroP). Behavioral estrus (blue bar) marks the height of female sexual receptivity (lordosis behavior), beginning after the peak of E2 and lasting until the morning of estrus.

Similar to E2, P4 is synthesized in the CNS. The synthesis of P4 within the central nervous system (neuroprogesterone or neuroP) is facilitated by E2, is sexually differentiated, and is a critical step in reproduction. NeuroP synthesis can be stimulated by E2 in the hypothalamus of female rodents, but not in males (73). This is mirrored physiologically, in that neuroP is critical for E2 positive feedback underlying the LH surge, which occurs in female, but not male, rodents. Originally, the adrenal gland was proposed to be the source of preovulatory P4 necessary for E2 positive feedback (74–77). However, removal of peripheral sources of P4 by ovx and adrenalectomy (adx) only blunts the E2-induced LH surge but does not eliminate it (73). In addition, peripheral levels of P4 peak after, not before, the LH surge (Figure 2). Importantly, when hypothalamic neuroP synthesis is blocked with a 3β-hydroxysteroid dehydrogenase (3β-HSD) inhibitor in ovx/adx rats, the LH surge is entirely eliminated (73). Central (3V) administration of P4 in these animals restores the LH surge. Further, in gonadally intact cycling female rats, blocking conversion of pregnenolone to P4 with aminoglutethimide (AGT, an inhibitor of P450scc enzyme) arrests estrous cyclicity and prevents the LH surge (78–81). Together, these results demonstrate that *de novo* synthesis of neuroP is a critical, downstream component of E2 positive feedback underlying the LH surge (see Figure 2).

In astrocytes, neuroP synthesis is mediated by membrane-initiated E2 signaling. Here, E2 acts through a membrane ERα–mGluR1a complex, which in turn activates phospholipase C and inositol trisphosphate, causing a release of intracellular calcium. E2 facilitation of neuroP synthesis within hypothalamic astrocytes is regulated by protein kinase A signaling, which causes phosphorylation and activation of steroid acute regulatory protein (StAR) and translocator protein (TSPO) (82). As in other cells, activated StAR and TSPO mediate the rate-limiting step of steroidogenesis, transport of cholesterol into the mitochondrion [(83); reviewed in Ref. (84); but see Ref. (85)].

E2-induced facilitation of neuroP synthesis *in vivo* correlates well with the sex- and age-specific ability to mount an LH surge. E2 does not facilitate neuroP synthesis in male hypothalamic astrocytes (86) and male rats are incapable of displaying an E2- or P4-induced LH surge (87). Similarly, the development of E2 positive feedback signaling that facilitates the LH surge occurs around the same time as the development of E2-induced neuroP synthesis in females. Prior to 28 days of age, a female rat will display an LH surge only in response to supra-physiological levels of E2, and lower levels will elicit a delayed surge. It is only after 28 days of age that a female rat will mount a temporally relevant LH surge in response to preovulatory levels of E2 (88). Observations from our laboratory indicate that prepubertal astrocytes do not synthesize neuroP in response to E2 administration (89), and maturation of these astrocytes *in vitro* does not induce the ability to respond to E2. In contrast, astrocytes harvested from adult female mice respond to E2 by synthesizing neuroP (90). This divergence of E2 responsivity between prepubertal and adult female astrocytes indicates a gain of function at the level of the hypothalamic astrocyte during puberty.

One possibility is that new cells born during puberty are responsive to E2 stimulation. Indeed, new populations of hypothalamic astrocytes are born in the AVPV during puberty, coinciding with the onset of E2 positive feedback and initial LH surges [Figure 4; (91)]. The appearance of these newborn astrocytes coincides with E2 responsivity of the hypothalamus. An intriguing hypothesis is that astrocytes born during and after puberty are the source of neuroP within the hypothalamus, thereby mediating estrogen positive feedback. Further experiments are needed to determine if newly born astrocytes in the hypothalamus are the source of neuroP that is critical for the LH surge.

## Non-Classical Steroid Signaling Regulates Reproduction

### Lordosis Behavior

Lordosis is a behavior indicating sexual receptivity that is displayed by female rodents under certain physiological conditions and in concert with appropriate sensory cues from a male. In gonadally intact female rodents, fluctuating levels of steroid hormones such as E2 and P4 induce changes in neuronal structure and signaling that allows for the expression of lordosis, indicating the ability of the female to engage in copulation (96). Sexual receptivity as measured by lordosis behavior can be elicited by a variety of steroid priming paradigms in ovx rodents [for review, see Ref. (97)]. The typical pattern of steroid priming involves daily low doses of estradiol benzoate for 1–4 days, followed by a dose of P4, which induces sexual receptivity 4 h later [e.g., Ref. (98)]. However, 3 V administration of P4 can induce lordosis behavior with 30 min [(99); reviewed in Ref. (3)]. While E2 alone is sufficient to induce lordosis behavior, larger doses of E2 are needed to induce receptivity [e.g., Ref. (100–102)]. It is becoming clear that different circuits are activated by E2 alone vs. E2 + P4 treatment (3). Finally, it should be appreciated that P4 has dual effects on lordosis: facilitation of E2 and subsequent termination of the behavior (103).

### Ovulation

#### E2-Positive Feedback

The neural mechanism underlying ovulation is a classic example of the interdependence of E2 and P4 in regulation of reproduction. Ovulation is triggered by surge release of GnRH from neurons in the hypothalamus, which in turn stimulate pituitary gonadotrophs to release LH. This surge of LH acts on the ovary, leading to release of a mature follicle. It has been well established that E2 affects GnRH neurons *via* both positive and negative feedback (104) albeit through distinct neuronal populations in the hypothalamus. This E2-regulated feedback is sex specific: males display only negative feedback in response to rising E2 levels (104–106), whereas females are capable of negative and positive feedback (107–110). This sex difference in LH surge capability is mirrored anatomically: male brains contain far fewer neurons in the AVPV, which is part of the rostral periventricular continuum of the third ventricle (RP3V) E2-positive feedback circuit in rodents; (111). In addition to this striking anatomical difference, there are other notable sex differences, such as E2-induced CREB phosphorylation in female, but not male, GnRH neurons (112). Ablation of this CREB signaling in GnRH neurons results in decreased fertility in females, but not males (113, 114). These *in vivo* results indicate an activation of GnRH neurons subsequent to E2 stimulation; they do not imply a direct action of E2 on GnRH neurons. While ERβ is expressed in GnRH neurons, the critical receptor, ERα, is not (115, 116). These results demonstrate the summation of central E2 actions at the level of GnRH neurons.

**Figure 3:**
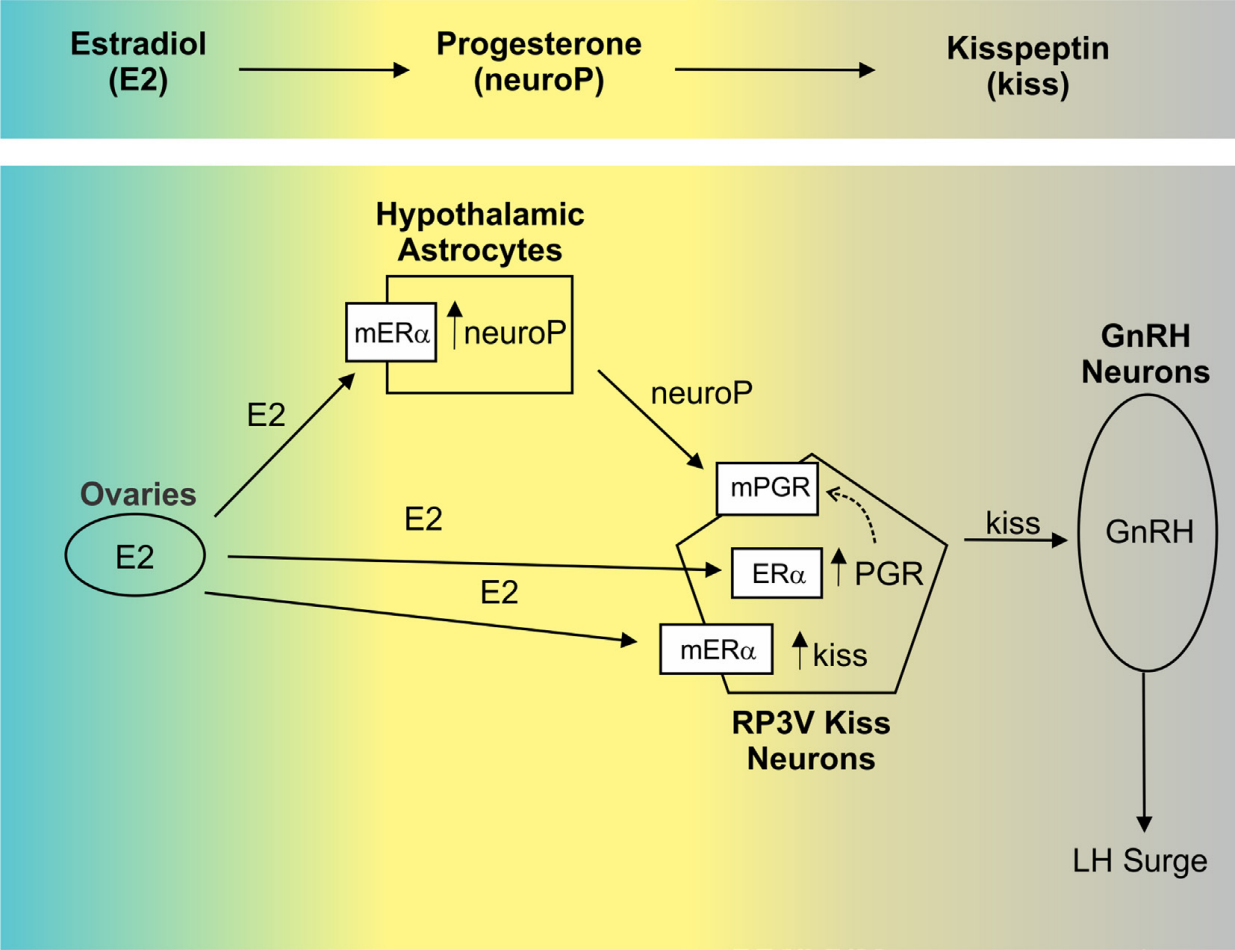
The order of hypothalamic signaling underlying the luteinizing hormone (LH) surge in the rodent. Estradiol (E2) upregulates progesterone receptor (PGR) in the anterior hypothalamus [e.g., Ref. (92, 93)], though the cell types in which this occurs was unknown. Recently, evidence has emerged indicating that E2-induced PGR occurs in RP3V kisspeptin (Kiss1) neurons (94) *via* membrane-localized ERα [mERα; (18)]. In this population, the neuropeptide Kiss1 is also induced by E2 (95). *In vitro*, E2 up regulation of Kiss1 was demonstrated to occur through activation of mERα, while PGR induction depended on classical signaling through nuclear estrogen receptor α (ERα) (18). PGR can then translocate to the membrane (dashed arrow). In addition to its actions in neurons, E2 also facilitates synthesis of progesterone (neuroP) within hypothalamic astrocytes *via* activation of mERα. While local synthesis of neuroP is critical to the LH surge [e.g., Ref. (73)] its cellular targets are not established. We hypothesize that neuroP acts locally within Kiss1 neurons (*via* mPGR) to augment E2 induction of Kiss1, which then activates gonadotropin-releasing hormone (GnRH) neurons. *In vitro*, immortalized Kiss1 neurons express PGR on the membrane and selective activation of PGR (using R5020) affects these cells within minutes supporting a functional role for membrane-localized PGR (mPGR in figure).

More recently, the sequence of hormone signaling within the hypothalamus has been uncovered: E2 stimulates neuroP synthesis and upregulates PGR (Figures 2 and 3). Indeed, that both ERα *and* PGR are necessary for reproductive function is revealed by studies using PRKO and ERα knockout mice. These models demonstrate the requirement of both PGR and ERα for successful reproduction. Female mice with ERα deletion do not exhibit estrous cycles and fail to reproduce, thereby implicating the need for ERα signaling in estrogen-positive feedback (117, 118). However, a more specific knockout, with altered DNA-binding domain in the estrogen response element (ERE) suggests that non-classical, extra-ERE E2 signaling accounts for a substantial component of E2-negative feedback, *via* regulation of GnRH neuron firing (119, 120). Together, these findings indicate clear roles for both classical, nuclear-initiated E2 signaling and non-classical, membrane-initiated E2 signaling. In addition to estrogens’ actions, several studies have indicated that PGR is also important. Namely, PRKO mice fail to mount LH surges in response to E2 treatment (121), as do rats following pharmacological or oligodeoxynucleotide blockade of P4/PGR signaling (122). These data support the dependence of E2 on downstream P4 signaling (Figure 3). The ERαKO mice demonstrate an interdependence of ERα and PGR, as PGR is markedly reduced (60%) in ERαKO mice compared to wild-type controls. Additionally, P4 treatments augment and advance the LH surge (123, 124).

**Figure 4:**
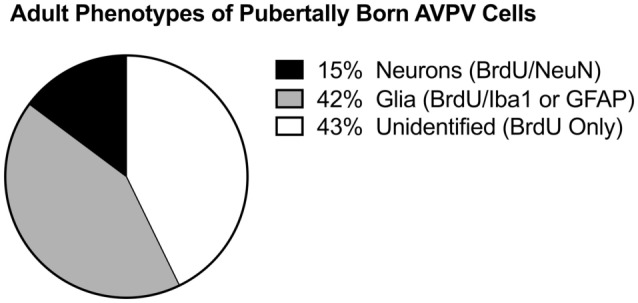
Pubertally born cells. Differentiation of pubertally born cells in the female rat anteroventral periventricular nucleus (AVPV). Female Sprague-Dawley rats were treated during puberty (P28–P56) with the thymidine analog, bromodeoxyuridine (BrdU), to label newly proliferated cells, then sacrificed in adulthood (P77) to examine survival and differentiation of these cells in the AVPV. Pie chart represents the mean proportion of pubertally born cells (BrdU-ir cells) that are glia (express Iba1-ir or GFAP-ir), neurons (express NeuN), or neither (unidentified, BrdU only). Figure modified and reproduced from Ref. (91), used with permission.

#### Hypothalamic Microcircuitry Controlling the LH Surge

Gonadotropin-releasing hormone neurons in the preoptic area of the hypothalamus release GnRH into the hypothalamic-hypophyseal portal system. Release of LH from the pituitary is temporally correlated with that of GnRH, including both low-level/pulsatile and surge release (125), making plasma LH measurements reliable indicators of central activity. Rising levels of E2 lead to surge release of GnRH and LH [estrogen-positive feedback; (126–128)]. This is not achieved through direct activation of GnRH neurons, as they do not express ERα, the critical ER mediating positive feedback (111). Instead, an upstream population of cells must mediate E2 feedback. This upstream population is now generally accepted to be neurons expressing kisspeptin (Kiss1) in the RP3V that includes the AVPV [reviewed in Ref. (129)]. Kiss1 neurons are the accepted site for E2-positive feedback based on the following evidence:

1. E2 induces Kiss1 expression in the RP3V *in vivo* [reviewed in Ref. (130)].
2. RP3V Kiss1 neurons express ERα (94, 95, 120, 131), the critical ER for positive feedback (111).
3. Kiss1 neurons express PGR, the PGR associated with positive feedback (132)
4. RP3V Kiss1 neurons project to GnRH neurons (111, 133), which express the Kiss1 receptor [GPR54/Kiss1R; (134)]. Importantly, this expression of GPR54 in GnRH neurons is critical for fertility [(135, 136); also see Ref. (137)].
5. Kiss1 is the most potent activator of GnRH neurons [e.g., Ref. (138)].

##### Cellular Targets

While rising E2 is a necessary precursor to the LH surge, preovulatory P4 signaling is similarly important [Figures 2 and 3; (73, 139–142)]. As previously described, the classical PGR is the critical receptor for multiple reproductive processes, including the LH surge. Likewise, Kiss1 signaling is requisite and is likely to be downstream of P4/neuroP signaling because Kiss1 neurons directly and potently stimulate GnRH neurons [e.g., Ref. (138)] and because Kiss1 consistently elicits an LH surge (143–145). However, the cellular targets mediating interactions between neuroP, and PGR are only now becoming clear. E2 stimulates PGR in the anterior hypothalamus and simultaneously induces neuroP production in astrocytes. From here, Kiss1 stimulates GnRH neurons, but it is not clear how Kiss1-induced GnRH activity is linked to both PGR upregulation and neuroP production. Our laboratory hypothesizes that Kiss1 neurons express the critical PGR receptors themselves, and E2 and neuroP signaling is integrated *within Kiss1 neurons*.

### E2-Induced PGR Expression in Kiss1 Neurons

Dual label immunohistochemistry studies show extensive PGR expression in arcuate nucleus [e.g., Ref. (146)] and in RP3V kisspeptin neurons (94, 147). Zhang and colleagues demonstrate co-localization of PGR in RP3V Kiss1 neurons (94), but only in tissue taken from animals in proestrus (high E2 levels). There was no co-localization in tissue taken from animals in diestrus (e.g., when levels of E2 are low), suggesting an E2-induced increase in PGR expression in RP3V Kiss1 neurons. Our laboratory has determined that PGR expression is increased by E2 in mHypoA51 cells, an *in vitro* model of RP3V Kiss1 neurons. mHypoA51s are immortalized cells derived from adult, female mouse hypothalamus that exhibit a robust increase in PGR expression following stimulation with E2 (18), consistent with the idea that E2 induces PGR in RP3V Kiss1 neurons *in vivo*.

#### PGR Expression in Kiss1 Neurons Is Critical for the LH Surge

We have shown that in immortalized Kiss1 neurons PGR does traffic to the cell membrane (41). Furthermore, this membrane-localized classical PGR is critical for full Kiss1 expression and possibly release. Stimulation with E2 induced PGR in these cells, after which a 5-min stimulation with R5020 (a selective PGR agonist) caused Erk1/2 phosphorylation (41). This was prevented with Src antagonist PP2, suggesting a PGR/Src interaction. PGR has been shown to associate with Src, although the physiological implications of this association are still emerging (42). Additionally, a number of other membrane progesterone-binding proteins are expressed in the hypothalamus, but their role in Kiss1 neuron function has not been elucidated. Kiss1 neurons *in vitro* (mHypoA51 cells) express mPRα and mPRβ; however, the expression of these receptors in mHypoA51 Kiss1 neurons is not modulated by E2, suggesting that their role may not be directly related to modulation of GnRH release (41). *In vivo*, mPRβ is induced by E2 in the anterior hypothalamus, though this may reflect upregulation in non-Kiss1 cells, as a number of cell types (neuronal and non-neuronal) are present in whole tissue (29). Therefore, while there is a clear role for PGR in the Kiss1 circuit governing ovulation, we have yet to determine how mPRs might be involved. It will also be interesting to learn the details of PGR signaling (i.e., to what extent membrane vs. classical signaling is involved, and which intracellular cascades are activated).

Two groups have recently demonstrated the necessity of PGR expression in Kiss1 neurons using a genetic knockout model. Kiss1 PRKO mice lack PGR expression specifically within Kiss1 cells and are, perhaps surprisingly, not infertile; however, they do not display the characteristic OVX + E2-induced LH surge (132, 148). This *in vivo* evidence solidifies the importance of classical PGR in Kiss1 neurons for the LH surge. These studies represent an important breakthrough in understanding the hypothalamic microcircuitry governing the LH surge. PGR within Kiss1 neurons represents the critical site for P4 action underlying the LH surge. Some questions remain, however: What subcellular localization is important in this process? Is membrane PGR involved? Moving forward, it will be interesting to learn the answers to these questions and to know the extent to which membrane P4 and neuroP signaling influence gonadotropin release in the female rodent.

## Conclusion

This review focuses on P4 membrane-initiated signaling regulating reproduction, especially within the CNS. Both *in vivo* and *in vitro* studies demonstrate that PGR expressed in Kiss1 neurons of the RP3V are important for E2-positive feedback on the LH surge. Kiss1/neurokinin B/dynorphin (KNDy) neurons in the ARH appear to mediate aspects of negative feedback [e.g., Ref. (149); see Ref. (150) for review]. Studies from our laboratory underscore the importance of E2-induced neuroP synthesis in female hypothalamic astrocytes and its role in activating the LH surge. In Kiss1 neurons, neuroP acts on PGRs to augment the E2 induction of Kiss1 expression and release (see Figure 3). Significantly, in prepubertal astrocytes, E2 is not capable of facilitating neuroP synthesis in females. Following puberty, female, but not male, hypothalamic astrocytes are highly responsive to E2. This shift appears to be due to a new population of astrocytes that are born during puberty and adulthood. What makes these pubertally born astrocytes competent to have E2-facilitated neuroP synthesis will require further experimentation. Interestingly, Kiss1 neurons, at least *in vitro*, express mPRs. While knockdown of the PGR abrogates the LH and reproduction, mPR function in Kiss1 neurons at this juncture remains unclear. PGRs are induced through direct nuclear action of E2, in our immortalized Kiss1 neuron model (18). A population of these PGRs is transported to the cell membrane and is stimulated by neuroP. The downstream signaling mechanisms appear to involve activation of Src, ERK1/2, and intracellular calcium. Thus, while there are many questions to address, it is now clear that membrane-initiated P4 signaling is involved in regulation of the LH surge—a cornerstone of P4 action. Similar to E2, it is likely that as research into P4 signaling continues, we will discover that this steroid can function in neurotransmitter-like fashion, and both classical and non-classical mechanisms of action are integrated to regulate critical reproductive functions, and many other neural phenomena in both PNS and CNS.

## Author Contributions

All authors (MM-S, LR, MM, and PM) contributed to this work through critical review of current and historical literature.

## Conflict of Interest Statement

The authors declare that the research was conducted in the absence of any commercial or financial relationships that could be construed as a potential conflict of interest.
